# Determining the Importance of the Stringent Response for Methicillin-Resistant *Staphylococcus aureus* Virulence In Vivo

**DOI:** 10.1093/infdis/jiaf421

**Published:** 2025-08-08

**Authors:** Naznin R Choudhury, Lucy Urwin, Bartłomiej Salamaga, Lynne R Prince, Stephen A Renshaw, Rebecca M Corrigan

**Affiliations:** School of Biosciences, University of Sheffield, Sheffield, United Kingdom; The Florey Institute, University of Sheffield, Sheffield, United Kingdom; The Florey Institute, University of Sheffield, Sheffield, United Kingdom; Division of Clinical Medicine, School of Medicine and Population Health, University of Sheffield, Sheffield, United Kingdom; School of Biosciences, University of Sheffield, Sheffield, United Kingdom; The Florey Institute, University of Sheffield, Sheffield, United Kingdom; Division of Clinical Medicine, School of Medicine and Population Health, University of Sheffield, Sheffield, United Kingdom; The Florey Institute, University of Sheffield, Sheffield, United Kingdom; Division of Clinical Medicine, School of Medicine and Population Health, University of Sheffield, Sheffield, United Kingdom; Bateson Centre, University of Sheffield, Sheffield, United Kingdom; School of Medicine, University College Dublin, Dublin, Ireland; Conway Institute, University College Dublin, Dublin, Ireland

**Keywords:** stringent response, (p)ppGpp, virulence, zebrafish, *Staphylococcus aureus*

## Abstract

The stringent response is a stress signaling pathway with links to bacterial virulence. This pathway is controlled by the nucleotide alarmone (p)ppGpp, produced in *Staphylococcus aureus* by 3 synthetase enzymes. Here, we used a panel of synthetase mutants to examine the importance of this signaling network for *S. aureus* survival and virulence in vivo. Using a zebrafish larval infection model, we observed that infection with a (p)ppGpp null strain attenuated virulence. Zebrafish myeloid cell depletion restored the virulence during systemic infection, indicating that (p)ppGpp is important for phagocyte-mediated immune evasion. Primary macrophages infection studies, followed by in vitro tolerance assays and RNA sequencing, revealed that (p)ppGpp is required to survive stressors found within the intracellular macrophage environment, with roles for each class of synthetase, and the linked transcription factor CodY, implicated. Taken together, these results define the importance of the stringent response and each class of synthetase for *S. aureus* infection.

## Teaser

The stringent response is a ubiquitous bacterial stress survival pathway. Here, we use a zebrafish embryo infection model, coupled with human macrophages studies, to show that this response is important for MRSA virulence by promoting bacterial adaptation to the phagolysosome.

**Figure 1.**
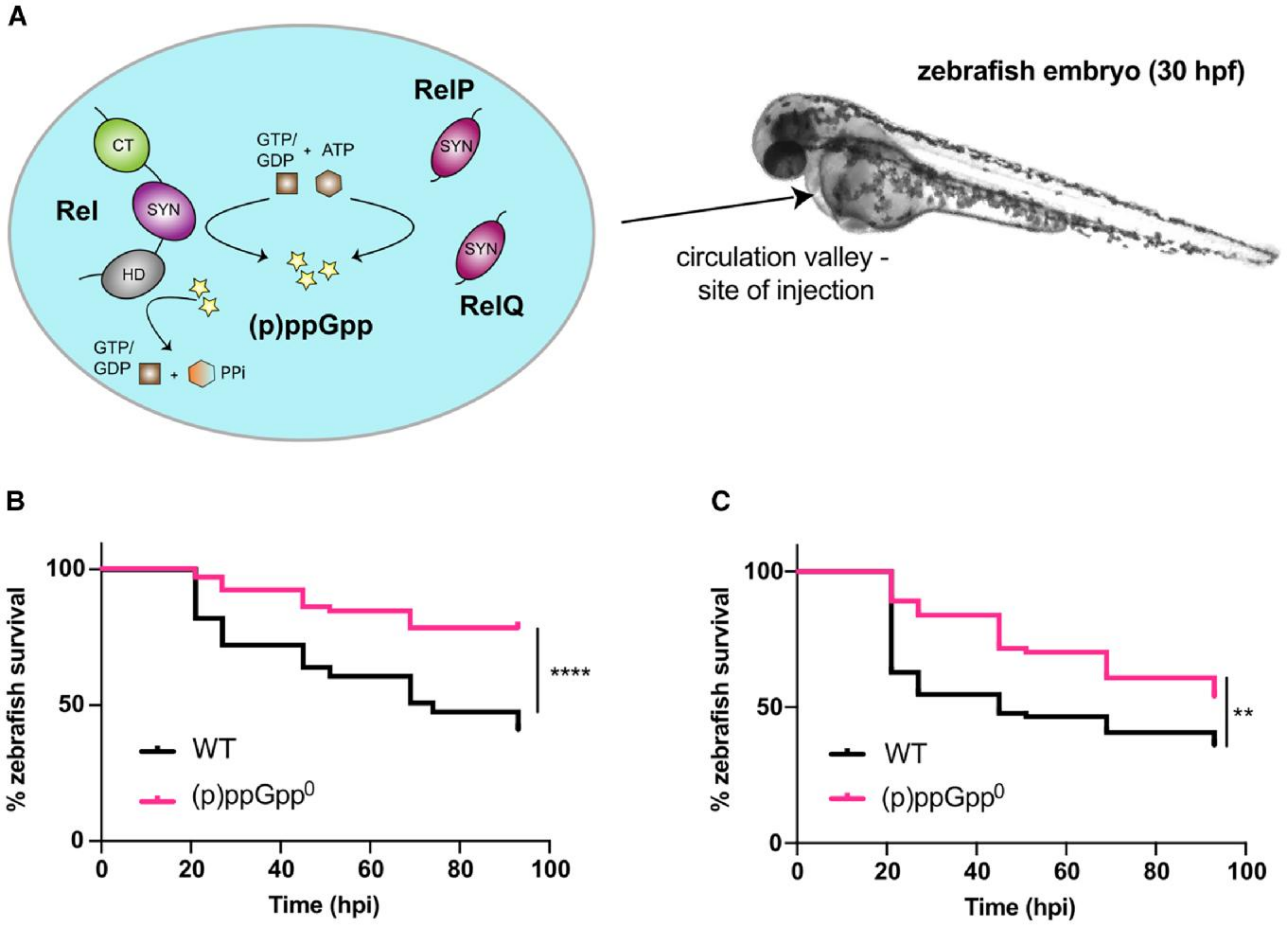
(p)ppGpp^0^  *Staphylococcus aureus* displays attenuated virulence in a systemic infection model. *A*, Schematic overview of the (p)ppGpp turnover enzymes in *S. aureus*. (p)ppGpp is produced by 3 enzymes, Rel, RelP, and RelQ via the SYN domain. Rel is also capable of hydrolyzing (p)ppGpp via the HD domain. Interactions between Rel and the ribosome occur via the CT domain and allow amino acid starvation to be sensed. *S. aureus* was injected into the yolk sac circulation valley of zebrafish embryos at 30 hpf. *B* and *C*, Survival of zebrafish larvae injected with *S. aureus* WT or (p)ppGpp^0^ grown to (*B*) exponential and (*C*) stationary phase. Doses of 3000–4000 colony-forming units of each strain were injected into the yolk sac circulation valley at 30 hpf to initiate a bloodstream infection. Survival was monitored until 93 hpi when the larvae reached 5.2 days postfertilization. Pairwise comparisons (log-rank [Mantel-Cox] test) were (*B*) (p)ppGpp^0^ versus WT, ****P < .0001; and (*C*) (p)ppGpp^0^ versus WT, **P = .0048. Experiments were performed in quadruplicate (*B*) and triplicate (*C*). Abbreviations: (p)ppGpp, guanosine tetraphosphate/guanosine pentaphosphate; (p)ppGpp^0^, (p)ppGpp null mutant; CT, C-terminal; hpf, hours postfertilization; HD, hydrolase; hpi, hours postinfection; SYN, synthetase; WT, wild type.

*Staphylococcus aureus* is a highly adaptable pathogen, with a large arsenal of virulence factors allowing colonization of diverse human tissues. During infection, bacteria face harsh conditions, including nutrient deprivation, pH fluctuations, and immune defenses. To cope, bacteria activate the stringent response, a conserved survival pathway coordinated by the nucleotides guanosine tetraphosphate (ppGpp) and guanosine pentaphosphate (pppGpp), collectively termed (p)ppGpp [1]. (p)ppGpp is produced by the RelA/SpoT homologue (RSH) protein family [2], with *S. aureus* encoding 3 such synthetases: the long RSH enzyme Rel, which also possesses hydrolase activity; and the monofunctional small alarmone synthetases (SAS) RelP and RelQ (Figure 1*A*) [3]. Once produced, (p)ppGpp triggers major cellular changes, inhibiting growth while upregulating stress adaptation, ultimately facilitating bacterial survival [4, 5].

Stringent response activation contributes to the pathogenicity of several bacterial species. For example, a *Salmonella* (p)ppGpp-null mutant, termed (p)ppGpp^0^, failed to replicate in mouse spleens after 5 days [6], while a *Mycobacterium tuberculosis rel* mutant had a 2-log reduction in lung and spleen tissues over 38 weeks, highlighting the importance of Rel for long-term viability [7]. In *S. aureus*, a methicillin-resistant (MRSA) *rel* mutant formed cutaneous abscesses over 13-times smaller than those of wild type (WT) [8]. Rel was also required for maintaining methicillin-sensitive *S. aureus* (MSSA) load in murine renal abscesses and for reducing mouse body weight [9]. This weight loss was dependent on the transcription factor CodY, which derepresses amino acid and virulence genes during stringent response activation [10]. While Rel is also crucial for *S. aureus* survival in polymorphonuclear leukocytes [11], the importance of the entire signaling system, including SAS enzymes, for staphylococcal virulence remains unclear.

Zebrafish (*Danio rerio*) are a valuable model for studying infections due to their high genetic similarity to humans [12]. As zebrafish have a functional innate immune system by 30 hours postfertilization (hpf) [13, 14], we previously used them to develop systemic infection models for studying *S. aureus* pathogenicity [15]. Here, we have used the versatility of zebrafish to establish the importance of (p)ppGpp for systemic *S. aureus* infection, extending previous findings relating to bacterial load in host organs to examine the contribution of the stringent response to host killing. We determine that both RSH and SAS synthetases contribute to *S. aureus* virulence. The attenuated phenotype of a (p)ppGpp^0^ mutant was partially myeloid cell dependent, as morpholino-mediated myeloid cell depletion restored virulence. We further show that (p)ppGpp was required for *S. aureus* survival within primary human macrophages, with in vitro studies highlighting its importance in phagolysosomal stress tolerance. Investigations into the mechanism of (p)ppGpp-controlled bacterial survival reveal a key role for CodY in tolerance to phagolysosomal stressors. Altogether, this work underscores the importance of (p)ppGpp and CodY for *S. aureus* growth, systemic infection, and host killing, whilst implicating both RSH and SAS enzymes in stress response in the host environment.

## METHODS

### Bacterial Strains and Culture Conditions

*Escherichia coli* strains were grown in Luria Bertani broth and *S. aureus* in tryptic soy broth at 37°C with shaking (200 rpm). Strains are listed in Supplementary Table 2 and primers in Supplementary Table 3.

### Zebrafish Husbandry and Embryo Infections

Zebrafish husbandry and injections were carried out as described previously and are outlined in the Supplementary Methods [15, 16].

### Isolation and Culture of Human Monocyte-Derived Macrophages

Peripheral blood mononuclear cells (PBMCs) were isolated from leukocyte cones (anonymized samples from consenting donors, UK NHS Blood and Transplant Service) by density centrifugation with Ficoll Paque Plus. The PBMC layer was extracted, platelets removed, and red blood cells lysed using ammonium-chloride-potassium buffer. PBMCs were resuspended in Roswell Park Memorial Institute (RPMI)-1640 medium containing 10% new-born calf serum, 1% L-glutamine, and 1% antibiotic-antimycotic solution, and seeded at 2 × 10^6^ cells/mL monocyte-derived macrophages (MDMs). After 24–48 hours, the medium was replaced with RPMI-1640 containing 10% fetal bovine serum (FBS), 1% L-glutamine, and 1% antibiotic-antimycotic solution, with media changes every 3–4 days to promote differentiation into M0 macrophages. On day 12, M0 macrophages were detached using accutase, pooled, and reseeded at 2 × 10^5^ cells/mL with RPMI-1640, 10% FBS, and 1% L-glutamine.

### Macrophage Infections

On day 13, MDMs were washed with Hanks' balanced salt solution containing calcium and magnesium and infected with bacteria at multiplicity of infection 10. Plates were centrifuged at low speed to synchronize infection and incubated at 37°C for 1 hour. Cells were washed twice with ice-cold PBS to remove nonadherent bacteria and halt internalization. To kill extracellular bacteria, 100 µg/mL gentamicin was added for 30 minutes at 37°C. For bacterial uptake measurements, a subpopulation of infected MDMs was washed twice with PBS and lysed with 2% saponin after gentamicin treatment. Intracellular bacteria were enumerated by plating. To measure bacterial killing, 100 µg/mL gentamicin was replaced with RPMI-1640 containing 4 µg/mL gentamicin and 0.8 µg/mL lysostaphin. Infected MDMs were incubated at 37°C until 3, 4.5, or 6 hours postinfection (hpi), and the gentamicin/lysostaphin removed. Infected cells were washed twice with PBS and intracellular bacteria enumerated.

### (p)ppGpp Quantification

(p)ppGpp was quantified as described previously and as outlined in the Supplementary Methods [17].

### Tolerance Assays

Cultures were diluted to optical density at 600 nm (OD_600_) 0.05 and grown to OD_600_ 0.35. Cells were washed twice in PBS prior to colony-forming unit (CFU) determination. Itaconic acid (20 mM) or H_2_O_2_ (100 mM from a 30% w/w stock) were then added and strains incubated at 37°C with shaking. CFUs were determined at 0.5 or 1 hour after addition and percent survival enumerated. Experiments were repeated up to 10 times due to variation in survival between biological replicates.

### RNA Extraction, Sequencing, and Analysis

RNA sequencing (RNA-seq) followed standard approaches as outlined in the Supplementary Methods [18].

## RESULTS

### (p)ppGpp Is Important for *S. aureus* Virulence

To assess the requirement of the stringent response for virulence, zebrafish embryos were infected with either the community-acquired MRSA strain JE2 (WT), or a (p)ppGpp^0^ mutant containing silent in-frame deletions in the 3 synthetase genes—*rel*, *relP,* and *relQ* [19]. Bacteria were injected into the bloodstream via the yolk sac circulation valley (Figure 1*A*), allowing entry into the heart and systemic dissemination [15]. A dose of approximately 3000–4000 CFU of WT caused 50% zebrafish mortality (Figure 1). In contrast, (p)ppGpp^0^ killed significantly fewer larvae. This occurred with both exponentially grown (*P* < .0001; Figure 1*B*) or stationary phase (*P* = .0048; Figure 1*C*) bacterial cultures, with no significant difference in mortality between the 2 growth phases. This confirms zebrafish larvae as a suitable model for studying lethal *S. aureus* infection and establishes a role for (p)ppGpp in *S. aureus* virulence.

**Figure 2.**
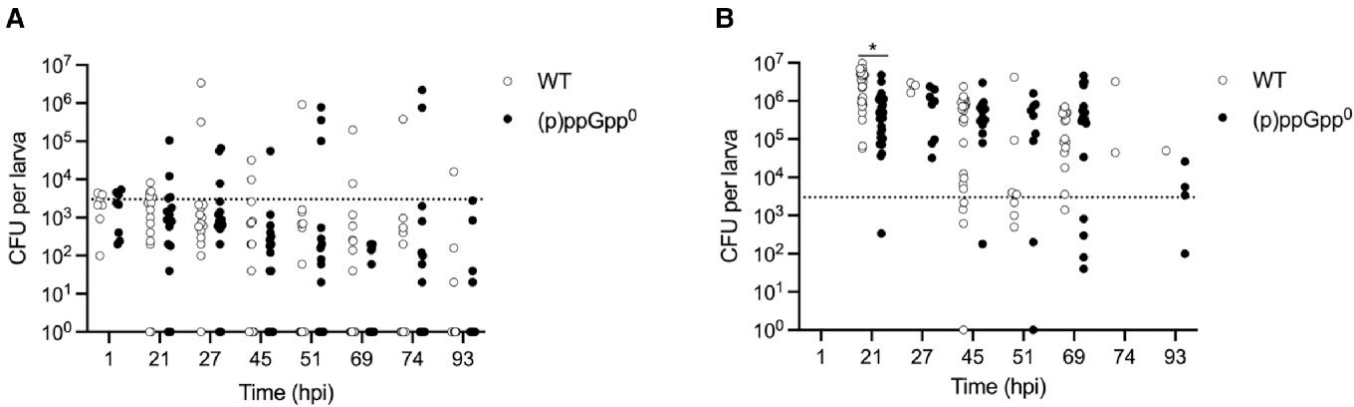
WT and (p)ppGpp^0^ mutant both replicate in vivo. Growth of *Staphylococcus aureus* WT and (p)ppGpp^0^ in (*A*) live and (*B*) dead zebrafish larvae after injection of 3000–4000 CFU (dotted line) into the bloodstream at 30 hours postfertilization. Zebrafish, 60–70, were injected with each strain, with 5 live larvae and any dead larvae taken at the specified time points for CFU/embryo determination. Survival was monitored until 93 hpi when the larvae reached 5.2 days postfertilization. The experiment was performed in biological triplicate with all 3 plotted. Abbreviations: (p)ppGpp^0^, guanosine tetraphosphate/guanosine pentaphosphate null mutant; CFU, colony-forming unit; hpi, hours postinfection; WT, wild type.

Although (p)ppGpp^0^ grows comparably to WT under nonstressed conditions in vitro [19], it may have a replication defect in vivo explaining its decreased ability to induce zebrafish mortality. To test this, the in vivo bacterial growth kinetics for both WT and (p)ppGpp^0^ were elucidated over the infection time course. Both strains were injected into zebrafish embryos and at each time point, up to 5 live larvae per strain (Figure 2*A*), and any dead larvae (Figure 2*B*) were homogenized and CFU/larva determined. In the dead embryos, bacterial loads increased from the initial inoculum of 10^3^ to between 10^5^ and 10^7^ CFU for both WT and mutant by 21 hpi (Figure 2*B*), with slight differences in CFU between WT and (p)ppGpp^0^ at 21 hpi. This demonstrates that both strains were able to replicate, although there were more dead larvae in the WT-infected population than with (p)ppGpp^0^. The live zebrafish either cleared the bacteria or maintained CFU counts at the initial inoculum level, with no significant differences in CFU between strains. Thus, while (p)ppGpp^0^ has attenuated virulence in vivo, this cannot be explained by an inability to replicate within the host.

### Both RSH and SAS Enzymes Contribute to *S. aureus* Virulence

**Figure 3.**
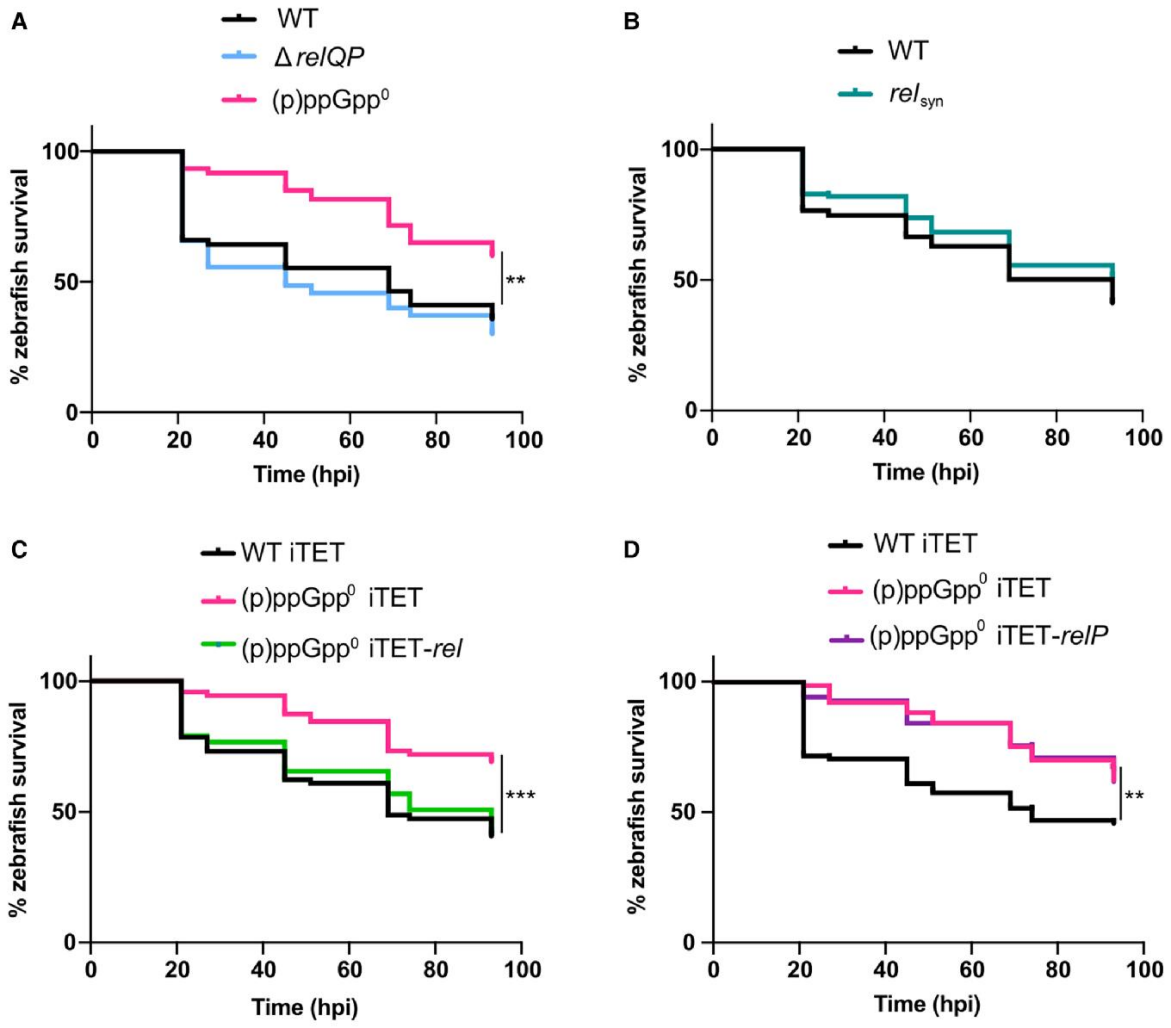
Both RSH and SAS (p)ppGpp synthetases contribute to *Staphylococcus aureus* virulence in a systemic zebrafish infection model. Survival of zebrafish larvae injected with *S. aureus* at 30 hours postfertilization. Survival was monitored until 93 hpi when the larvae reached 5.2 days postfertilization. *A*, Injection of WT, (p)ppGpp^0^, and Δ*relQP* (dose 3000–4000 CFU). *B*, Injection of WT and *rel_syn_* (dose 1500 CFU). *C*, Injection of WT iTET, (p)ppGpp^0^ iTET, and (p)ppGpp^0^ iTET-*rel* (dose 3000–4000 CFU). *D*, Injection of WT iTET, (p)ppGpp^0^ iTET, and (p)ppGpp^0^ iTET-*relP* (dose 3000–4000 CFU). *A*–*D*, Statistical significance was determined by log-rank (Mantel-Cox) test: **P < .01, ***P < .001. *A*–*C*, Experiments were performed in triplicate, while (*D*) was performed in quadruplicate. Abbreviations: (p)ppGpp, guanosine tetraphosphate/guanosine pentaphosphate; (p)ppGpp^0^, (p)ppGpp null mutant; CFU, colony-forming unit; hpi, hours postinfection; RSH, RelA/SpoT homologue; SAS, small alarmone synthetase; WT, wild type.

To understand the contribution of RSH versus SAS enzymes to infection, the virulence of WT and (p)ppGpp^0^ were first compared to Δ*relQP*, a strain with in-frame deletions of both SAS enzymes. Survival curves revealed Δ*relQP* killed larvae similarly to WT (Figure 3*A*), indicating that Rel alone is sufficient for virulence. We next tested whether SAS enzymes alone (without Rel activity) affected virulence. We used a Rel mutant strain (Rel_syn_) where 3 conserved amino acids in the synthetase domain (Y308/Q309/S310) are deleted, inactivating synthesis but retaining the hydrolase function, which is essential in strains encoding RelP and RelQ to prevent toxic accumulation of (p)ppGpp [9, 17]. Rel_syn_ and WT showed similar levels of zebrafish killing (Figure 3*B*). This suggests that while Rel alone is sufficient for WT levels of mortality (Figure 3*A*), (p)ppGpp produced by RelP and/or RelQ in the Rel_syn_ strain is enough to compensate for a lack of Rel.

To investigate further, (p)ppGpp^0^ was complemented with either the full-length *rel* from the integrative vector pCL55iTETr862 (iTET) or with the single SAS enzyme *relP* (Figure 3*C* and 3*D*). Note, iTET is leaky and uninduced expression of Rel or RelP can fully complement growth under starvation conditions (Supplementary Figure 1). While expression of Rel restored larval killing to WT levels (Figure 3*C*), complementation with the single SAS enzyme *relP* did not (Figure 3*D*). This confirms the importance of Rel in vivo, as has been reported previously [8, 9], but also indicates that either RelQ, or the presence of both SAS enzymes, is required for virulence. Altogether, these results support a general role for (p)ppGpp in *S. aureus* virulence that cannot be attributed to a specific class of synthetase.

### The Attenuated Virulence of (p)ppGpp^0^ Is Myeloid Cell Dependent

**Figure 4.**
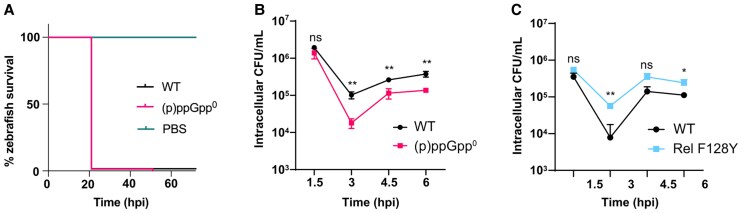
Macrophages are required to control *Staphylococcus aureus* infection in a (p)ppGpp-dependent manner. *A*, Survival of Pu.1 knockdown zebrafish larvae injected with PBS, WT, or (p)ppGpp^0^ at doses of 3000–4000 CFU at 30 hours postfertilization into the circulation valley. The Pu.1 morpholino, 1 pmol, was injected into the yolk of 1-cell stage embryos. Survival was monitored until 93 hpi when the larvae reached 5.2 days postfertilization. The experiment was performed in triplicate. Statistical significance was determined by log-rank (Mantel-Cox) test: ns between WT and (p)ppGpp^0^, *P* = .5275. *B* and *C*, Intracellular survival of WT *S. aureus* within primary human macrophages, as compared to (*B*) (p)ppGpp^0^ and (*C*) (p)ppGpp overexpression strain, Rel F128Y. MDMs were infected with bacteria at multiplicity of infection of 10 for 1 hour, before addition of 100 µg/mL gentamicin to kill extracellular bacteria. Infected MDMs were lysed at 1.5, 3, 4.5, or 6 hpi and plated to measure intracellular CFU/mL. Experiments were repeated 4 times for (*B*) (p)ppGpp^0^ (see also Supplementary Figure 2) and 3 times for (*C*) Rel F128Y (Supplementary Figure 4) using MDMs from different donors. Data from 1 representative donor are presented for each experiment. For each donor MDM population, 2 technical repeats were performed. Statistical significance was determined within each MDM donor population by unpaired *t* test: ns, *P* > .05; *P < .05; **P < .01. Abbreviations: (p)ppGpp, guanosine tetraphosphate/guanosine pentaphosphate; (p)ppGpp^0^, (p)ppGpp null mutant; CFU, colony-forming unit; hpi, hours postinfection; MDM, monocyte-derived macrophage; ns, not significant; PBS, phosphate-buffered saline; WT, wild type.

During early development, zebrafish larvae largely rely on myeloid cells to protect against infection [20], although epithelial cell-mediated phagocytosis also contributes to defense [21]. To determine the contribution of myeloid cells in controlling (p)ppGpp^0^ virulence, WT and mutant strains were injected into embryos with depleted myeloid cells. Here, a morpholino-modified antisense oligonucleotide was employed to transiently knockdown *pu.1*, a transcription factor needed for pluripotent hematopoietic stem cell differentiation [22], which delayed the appearance of macrophages and neutrophils from 25 to 48 hpf, and 30 to 36 hpf, respectively [13, 14, 23, 24]. At the 1-cell developmental stage, embryos were injected with morpholino into the yolk sac, followed by injection of either WT or (p)ppGpp^0^ at 30 hpf into the circulation valley. Myeloid cell depletion resulted in 100% larval mortality within 24 hpi and, crucially, restored the virulence of (p)ppGpp^0^ to WT levels (Figure 4*A*). This indicates that myeloid cells are critical for controlling infection and that (p)ppGpp is required to enable *S. aureus* evasion of phagocytic killing.

### (p)ppGpp Is Required for *S. aureus* Survival Within Human Macrophages

Previous work highlights the importance of Rel for *S. aureus* survival within polymorphonuclear leukocytes [11]. Building on our findings that both RSH and SAS synthetases contribute to virulence, and that myeloid cell depletion restores (p)ppGpp^0^ virulence to WT, we wished to further examine the link between (p)ppGpp and *S. aureus* survival within phagocytes. Human macrophages were infected with either WT or (p)ppGpp^0^ and intracellular survival studied. We observed that both strains are initially killed, before adaptation and subsequent proliferation; however, (p)ppGpp^0^ displayed significantly reduced survival in macrophages compared to WT (Figure 4*B* and Supplementary Figure 2).

Clinical strains with elevated (p)ppGpp levels have been associated with persistent infections [25–28]. One such *S. aureus* strain isolated from a persistent bacteremic infection contained an F128Y substitution in the Rel hydrolase domain, causing constitutive, partial stringent response activation [26, 27]. We confirmed that the introduction of F128Y into our MRSA background elevated (p)ppGpp production, with basal levels increased 1.5-fold (Supplementary Figure 3) and then examined the impact of this on *S. aureus* survival within macrophages. Interestingly, we observed increased survival of Rel F128Y (Figure 4*C* and Supplementary Figure 4), further supporting a role for (p)ppGpp in intracellular survival.

### (p)ppGpp^0^ Is More Susceptible to Stress Conditions Found Within Macrophages

Upon infection, *S. aureus* is exposed to phagolysosomal stressors in macrophages, including low pH and reactive oxygen/nitrogen (ROS/RNS) species [29]. Acidification is mainly driven by the proton-pumping v-ATPase, although metabolites like itaconic acid also contribute.

**Figure 5.**
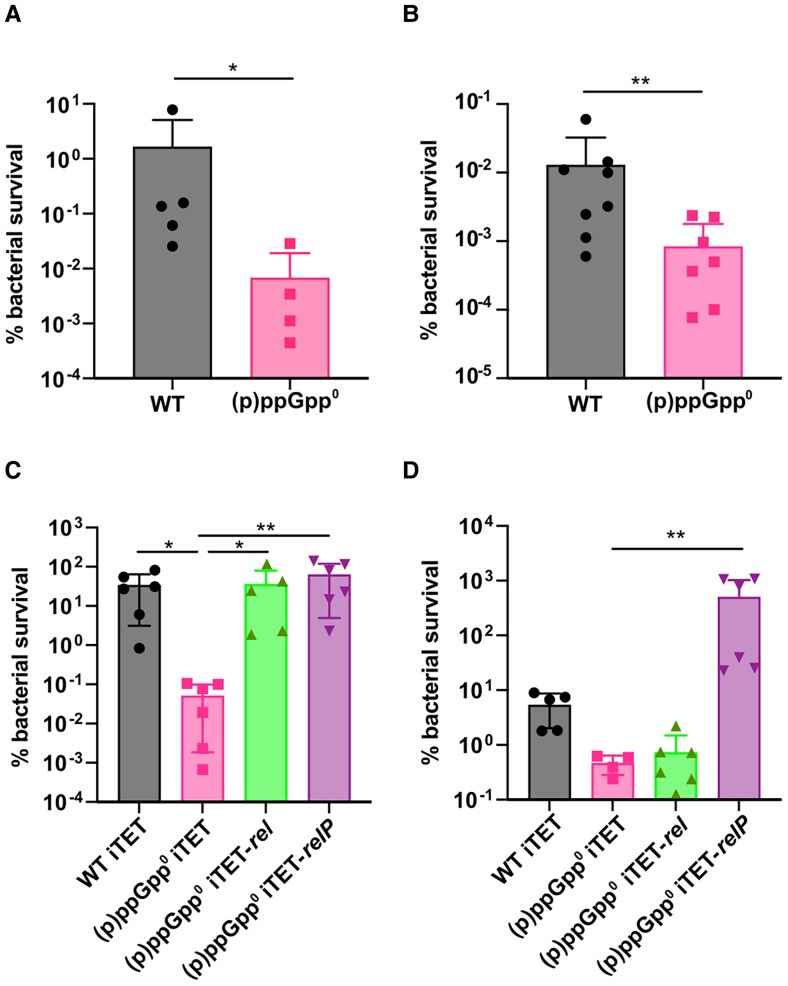
The (p)ppGpp^0^ mutant is less tolerant to stressors found within professional phagocytes. WT and (p)ppGpp^0^ were grown to OD_600_ 0.35 in tryptic soy broth. Cells were washed twice in PBS and exposed to (*A*) 100 mM H_2_O_2_ or (*B*) 20 mM itaconic acid for 1 hour at 37°C before CFU determination. Statistical analysis performed using Mann-Whitney test. *C* and *D*, WT iTET, (p)ppGpp^0^ iTET, (p)ppGpp^0^ iTET-*rel*, and (p)ppGpp^0^ iTET-*relP* were grown to an OD_600_ of 0.35. Cultures were washed twice in PBS and exposed to (*C*) 100 mM H_2_O_2_ or (*D*) 20 mM itaconic acid for 30 minutes at 37°C and the CFU/mL was determined. Percentage bacterial survival with mean and standard deviation are plotted. Statistical analysis performed using a Kruskal-Wallis test followed by a Dunn multiple comparison test. *P < .05, **P< .01. Abbreviations: (p)ppGpp^0^, guanosine tetraphosphate/guanosine pentaphosphate null mutant; CFU, colony-forming unit; OD_600_, optical density at 600 nm; PBS, phosphate-buffered saline; WT, wild type.

Previously, an MSSA (p)ppGpp^0^ mutant exhibited susceptibility to H_2_O_2_ [5], while an MRSA (p)ppGpp^0^ mutant displayed reduced tolerance to HOCl [30], implicating the stringent response in phagolysosomal stress resistance. Here, WT and (p)ppGpp^0^ were exposed to H_2_O_2_ and itaconic acid, revealing that the mutant was 1–2 log more susceptible to both stresses (Figure 5*A* and 5*B*). To examine roles for each synthetase class in combatting external stressors, (p)ppGpp^0^ was complemented with either the RSH Rel or SAS RelP. While expression of RelP was unable to restore virulence in our zebrafish infection model (Figure 3*D*), it was sufficient to restore tolerance to both ROS and pH stress in vitro (Figure 5*C* and 5*D*). Meanwhile, expression of Rel conferred tolerance to ROS only (Figure 5*C*). These findings are in keeping with prior reports linking SAS enzymes to pH stress [3, 31], and Rel to combatting ROS, supporting distinct stress-response roles for different synthetases.

### Deleting *codY* Eliminates (p)ppGpp^0^ Survival Defects In Vitro but not In Vivo

Under nutrient-rich conditions, CodY represses genes involved in nutrient uptake and virulence, with GTP and branched-chain amino acids acting as CodY cofactors [10]. During the stringent response, rising (p)ppGpp reduces GTP levels [4], derepressing the CodY regulon to cope with the changing environment. As GTP levels are increased in (p)ppGpp^0^ [19], we hypothesized that sustained CodY repression contributes to the reduced virulence.

**Figure 6.**
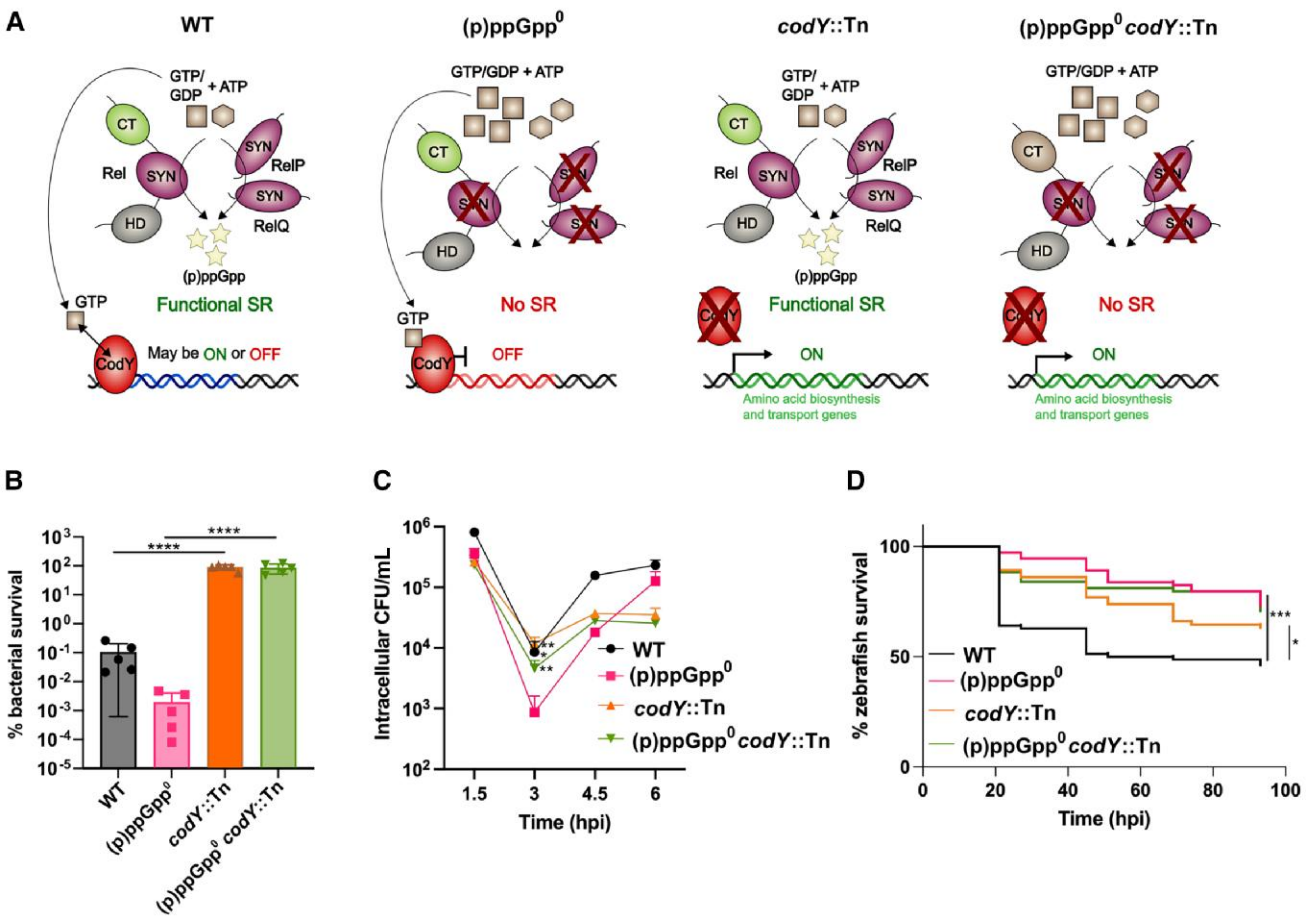
Deleting *codY* restores survival in vitro. *A*, Schematic representation of the indirect link between activation of the stringent response and derepression of the CodY regulon, as mediated by intracellular GTP levels. *B*, Susceptibility of WT, (p)ppGpp^0^, *codY*::Tn, and (p)ppGpp^0^  *codY*::Tn to 100 mM H_2_O_2_. Percentage bacterial survival with mean and standard deviation are plotted. Statistical analysis was performed using 1-way ANOVA with Tukey multiple comparisons test. *C*, Intracellular survival of WT, (p)ppGpp^0^, *codY*::Tn, and (p)ppGpp^0^  *codY*::Tn strains within primary human macrophages. MDMs were infected with bacteria at multiplicity of infection 10 for 1 hour, before addition of 100 µg/mL gentamicin to kill extracellular bacteria. Infected MDMs were lysed at 1.5, 3, 4.5, or 6 hpi and plated to measure intracellular CFU/mL. Infection assays were repeated 3 times (see also Supplementary Figure 6) using MDMs from 3 different donors. Data from 1 representative donor are shown. For each donor MDM population, 2 technical repeats were performed. Statistical significance was determined within each MDM donor population by unpaired *t* test. *D*, Survival of zebrafish larvae injected with WT, (p)ppGpp^0^, *codY*::Tn, and (p)ppGpp^0^  *codY*::Tn at doses of 3000–4000 CFU at 30 hours postfertilization into the circulation. Survival was monitored until 93 hpi when the larvae reached 5.2 days postfertilization. Statistical significance was determined by log-rank (Mantel-Cox) test. The experiment was performed in triplicate. *P < .05, **P < .01, ***P < .001, ****P < .0001. Abbreviations: (p)ppGpp^0^, guanosine tetraphosphate/guanosine pentaphosphate null mutant; CFU, colony-forming unit; CT, C-terminal; HD, hydrolase; hpi, hours postinfection; MDM, monocyte-derived macrophage; SR, stringent response; SYN, synthetase; WT, wild type.

To investigate this, *codY* was deleted in both WT and (p)ppGpp^0^ (Figure 6*A*) and strains exposed to itaconic acid or H_2_O_2_. In both backgrounds, deleting *codY* increased tolerance (Figure 6*B* and Supplementary Figure 5). To examine the importance of (p)ppGpp-controlled regulation of CodY during infection, primary macrophages were infected with WT, (p)ppGpp^0^, and corresponding *codY* mutants. While there was no difference between the WT and *codY* mutant, deleting *codY* in a (p)ppGpp^0^ background restored survival to WT levels (Figure 6*C*), highlighting the importance of CodY derepression in phagocytes. To examine the importance of CodY during systemic infection, zebrafish embryos were injected with the *codY* mutants. In contrast to the in vitro results, deleting *codY* did not rescue the attenuated (p)ppGpp virulence phenotype, with the *codY* mutant itself displaying a virulence defect (Figure 6*D*). This suggests that while inducing the CodY regulon is sufficient for enabling bacterial survival in macrophages, it is not enough to restore virulence during systemic infection and suggests that processes regulated by (p)ppGpp independently of CodY are also important for *S. aureus* virulence.

### (p)ppGpp Alters Gene Expression During ROS and pH Stress

**Figure 7.**
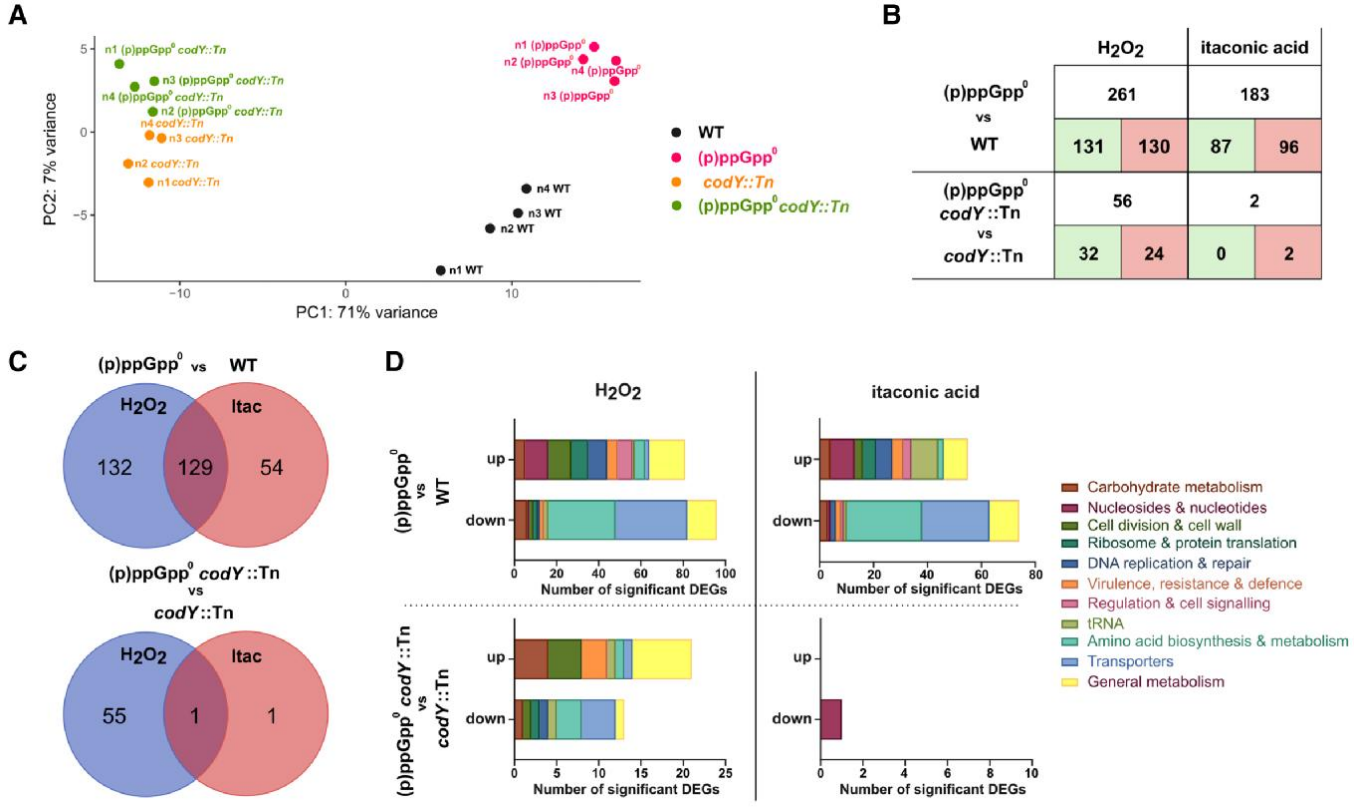
RNA-seq shows (p)ppGpp regulates hundreds of genes in response to reactive oxygen species and pH stress, predominantly via derepression of the CodY regulon. RNA was extracted from bacterial cultures of WT, (p)ppGpp^0^, *codY*::Tn, or (p)ppGpp^0^  *codY*::Tn that had been exposed to either 100 mM H_2_O_2_ or 20 mM itaconic acid for 10 minutes. Four biological replicates were performed per experimental condition. *A*, PCA of RNA-seq count data collected under H_2_O_2_-stress conditions. PCA plot was generated by DESeq2 on the Galaxy web platform. *B*, Number of significant DEGs in CodY-dependent (WT vs [p]ppGpp^0^) and CodY-independent backgrounds (*codY*::Tn vs [p]ppGpp^0^  *codY*::Tn), following H_2_O_2_ or itaconic acid stress. Green indicates upregulated genes, red represents downregulated genes. *C*, Overlaps between H_2_O_2_ or itaconic acid-induced stress responses in CodY-dependent and CodY-independent backgrounds. *D*, Functional categorization of significant DEGs using the KEGG mapper search tool. Hypothetical proteins are excluded for clarity. Abbreviations: (p)ppGpp, guanosine tetraphosphate/guanosine pentaphosphate; (p)ppGpp^0^, (p)ppGpp null mutant; DEG, differentially expressed gene; Itac, itaconic acid; PCA, principal component analysis; RNA-seq, RNA sequencing; WT, wild type.

We next sought to investigate the (p)ppGpp-controlled transcriptional changes that facilitate increased *S. aureus* tolerance to ROS and pH stress. Here, cultures were treated with either H_2_O_2_ or itaconic acid and the transcriptome analyzed. As (p)ppGpp alters gene expression via both CodY-dependent and -independent pathways [5, 11], RNA-seq was performed in CodY-positive (WT vs [p]ppGpp^0^) and CodY-negative (*codY*::Tn vs [p]ppGpp^0^  *codY*::Tn) strains. Principal component analysis showed strain-specific clustering under both stress conditions (Figure 7*A* and Supplementary Figure 7), with CodY accounting for most transcriptomic variation (Figure 7*B*).

RNA-seq identified 261 and 183 significant differentially expressed genes (DEGs) in (p)ppGpp^0^ compared to WT following H_2_O_2_ and itaconic acid stress, respectively (Figure 7*B* and Supplementary Table 1), underscoring the importance of (p)ppGpp in stress adaptation. Nearly half (129 DEGs) were shared between stresses (Figure 7*C*). Functional categorization of these common DEGs using the KEGG database [32] highlighted that (p)ppGpp promotes amino acid biosynthesis and transport, whilst repressing purine and carbon metabolism in WT strains in response to both stresses.

Under H_2_O_2_ stress, 79% of (p)ppGpp-regulated genes were CodY dependent, while nearly all were CodY dependent for itaconic acid stress (Figure 7*B*). KEGG analysis revealed that in these conditions, CodY regulates transporter and amino acid biosynthesis genes (Figure 7*D*), consistent with (p)ppGpp-driven derepression of nutrient acquisition via CodY [10]. Functional responses were similar between H_2_O_2_ and itaconic acid stress conditions in CodY-positive strains (compare [p]ppGpp^0^ vs WT; Figure 7*D*); however, several genes were regulated in a CodY-independent manner for H_2_O_2_ stress (Figure 7*D*). Here, loss of (p)ppGpp-dependent regulation led to the upregulation of general metabolism, including carbon metabolism, suggesting a direct role for (p)ppGpp in repressing carbon metabolism during oxidative stress. These findings confirm that while (p)ppGpp broadly regulates transcription via CodY, it also exerts CodY-independent effects, specifically under ROS stress.

## DISCUSSION

During infection, adverse environmental conditions trigger (p)ppGpp production, enabling *S. aureus* adaptation and survival. Previous studies have linked this response to antibiotic resistance [26, 33, 34], immune evasion [11], oxidative stress resistance [5, 30], and murine pyelonephritis [9]. Thus, this study aimed to systematically assess the role of (p)ppGpp in *S. aureus* virulence.

Our data reveal that (p)ppGpp is needed for systemic staphylococcal infection (Figure 1). During nutrient starvation, (p)ppGpp production and subsequent GTP depletion are required for derepression of amino acid transport and synthesis genes via CodY [10]. We thus hypothesized that the absence of (p)ppGpp could reduce nutrient acquisition leading to a growth defect in vivo and explaining the decreased zebrafish mortality. However, this was not the case, as both WT and (p)ppGpp^0^ proliferated similarly (Figure 2). This aligns with prior findings showing reduced cutaneous abscess formation by an *S. aureus rel* mutant, despite comparable CFU loads per abscess for WT and mutant [8]. In contrast, lower bacterial loads were observed in murine kidneys infected with an MSSA *rel*_syn_ mutant [9], suggesting tissue-specific effects.

By transiently depleting myeloid cells in zebrafish embryos, we observed that (p)ppGpp^0^ virulence was restored (Figure 4*A*), supporting the idea that phagocytes are required for controlling *S. aureus* infection. However, we note that differences in killing may still be observed by using lower inoculum or by monitoring killing before 21 hpi. Additionally, as neutrophils are the most abundant circulating phagocyte [35] and are often the first immune cells to infiltrate an infection site, further studies on these cells are needed to better understand (p)ppGpp's contribution to immune evasion.

While (p)ppGpp has been previously linked to ROS survival [28], we show that it is also essential for tolerating itaconic acid, which may aid survival within macrophages. In vivo, itaconic acid has functions in addition to modulating pH. It hinders bacterial growth by inhibiting isocitrate lyase, a key enzyme in the glyoxylate shunt [36–38]. Additionally, itaconate reduces inflammation during *S. aureus* ocular infection by modulating NRF2/HO1 signaling and suppressing the NLRP3 inflammasome [39]. Further research is needed to clarify how (p)ppGpp influences these anti-inflammatory effects.

This study demonstrates that both RSH and SAS synthetases contribute to virulence. The importance of Rel, revealed by Geiger and colleagues [9, 11], is corroborated by our studies showing that both *rel* expression in (p)ppGpp^0^ (Figure 3*C*), or the presence of *rel* alone in Δ*relQP* (Figure 3*A*), is associated with WT levels of virulence. We further show that RelP and/or RelQ were sufficient for virulence in the absence of Rel (Figure 3*B*). Given that RelP and RelQ are transcriptionally activated by cell wall and pH stress [4], and the acidic environment encountered once phagocytosed, RelP/RelQ likely support in vivo survival by producing (p)ppGpp in response to these signals. Further studies are necessary to elucidate whether RelQ alone is sufficient for virulence, or whether the combined presence of both SAS enzymes is needed.

(p)ppGpp functioning is closely linked to CodY. Previous studies reported that while a *codY* deletion alone did not affect *S. aureus* virulence, inactivating *codY* in an *S. aureus rel* mutant enhanced survival in phagocytes, which also occurred in *Listeria monocytogenes* [9, 40]. Consistent with this, both *codY*::Tn and (p)ppGpp^0^  *codY*::Tn showed increased resistance to itaconic acid, H₂O₂, and improved survival within macrophages (Figure 6). Interestingly, deleting *codY* did not increase virulence in vivo (Figure 6*D*). This is noteworthy, as CodY represses many virulence-associated genes, including the virulence regulator *agr* [41, 42]. Previous studies report that following phagocytosis, (p)ppGpp is required for the upregulation of the cytotoxic phenol-soluble modulins [11]. We speculate that reduced phenol-soluble modulin upregulation could account for reduced zebrafish killing by (p)ppGpp^0^, as bacteria may have reduced phagolysosome escape. As the (p)ppGpp and CodY regulatory networks are complex and intertwined, investigations into the expression of the CodY regulon during infection of zebrafish are necessary to further understand this.

Given the importance of CodY for *S. aureus* tolerance, we sought to characterize stringently regulated transcriptomic changes induced by H_2_O_2_ or itaconic acid. RNA-seq revealed that (p)ppGpp influences hundreds of genes, predominantly through CodY (Figure 7), where we observed significant transporter and amino acid biosynthesis gene downregulation in (p)ppGpp^0^. CodY is known to be required during acid stress in *Streptococcus* species [43], a finding recapitulated here (Supplementary Figure 5). In streptococci, the DNA-binding capacity of CodY diminishes at low pH, leading to amino acid synthesis gene derepression [43]. This may be required as acid tolerance can induce branched-chain amino acid catabolism for ATP generation [44, 45]. CodY-independent transcriptional changes were also observed, mostly under H_2_O_2_ stress. Here we observed that (p)ppGpp was crucial for the downregulation of genes involved in carbon metabolism. This metabolic suppression may help to reduce ATP generation and ROS production under stress, promoting survival.

Altogether, this study demonstrates that (p)ppGpp produced by both RSH and SAS synthetases contributes to *S. aureus* virulence, likely aiding survival and escape from phagolysosomal stress. We further show that zebrafish infection models are versatile tools for studying the importance of nucleotide signaling in living models. With the recent development of biosensors for (p)ppGpp, future work could now focus on using embryos to track nucleotide production during infection in real time.

## Supplementary Material

jiaf421_Supplementary_Data

## Supplementary Data

Supplementary materials are available at *The Journal of Infectious Diseases* online (http://jid.oxfordjournals.org/). Supplementary materials consist of data provided by the author that are published to benefit the reader. The posted materials are not copyedited. The contents of all supplementary data are the sole responsibility of the authors. Questions or messages regarding errors should be addressed to the author.
