# Supplementary material for: Determining the Importance of the Stringent Response for Methicillin-Resistant Staphylococcus aureus Virulence In Vivo
Source: J Infect Dis. 2025 Aug 8;232(5):e753–64. doi: 10.1093/infdis/jiaf421 (PMC12614963; doi:10.1093/infdis/jiaf421)
Supplement: jiaf421_Supplementary_Data [file jiaf421_supplementary_data.zip › SUPPLEMENTAL.pdf]

SUPPLEMENTAL

Supplementary Methods

Plasmid and strain construction. Plasmids pCL55iTETr862-*rel* and pCL55iTETr862-*relP* were constructed by amplifying the respective genes using the primers listed in Table S3. The resulting PCR products were digested and cloned into pCL55iTETr862 that had been digested with the same enzymes. All plasmids were initially transformed into *E. coli* strain XL1-Blue and sequences of all inserts were verified by fluorescence automated sequencing by Eurofins. pCL55iTETr862 plasmids were electroporated into RN4220 before being phage transduced into JE2 using Φ85. Φ85 was also used to move the *codY*::Tn transposon mutation into JE2 and JE2 (p)ppGpp^0.

Zebrafish strains and husbandry. Up to 5 days post fertilization (dpf) zebrafish are not protected under the Animals (Scientific Procedures) Act 1986. However, all work was carried out according to the stipulations set out in Project License P1A4A7A5E. London wildtype (LWT) strains were used for all zebrafish experiments. Adult zebrafish were maintained by staff at the University of Sheffield Bateson Centre Zebrafish Facility according to established standards [1]. Adult fish were kept at 28°C in a 14 hr/10 hr light/dark regime. Embryos/larvae were incubated at 28°C in E3 medium (0.5 mM NaCl, 17 μM KCl, 33 μM $CaCl_2$, 33 μM $MgSO_4$, 0.00005% methylene blue).

Zebrafish embryo microinjections. At approximately 30 hours post fertilisation (hpf), LWT zebrafish embryos were dechorionated and anaesthetised by immersion in 0.02% w/v buffered tricaine. The embryos were embedded in 3% w/v methylcellulose on a glass slide. 1 nl of bacterial suspensions were injected into the yolk sac circulation valley of the ≥ 30 embryos per condition using a pneumatic micropump (World Precision Instruments PV820), a

micromanipulator (WPI) and a dissecting microscope. Following injection, embryos were recovered in fresh E3 and placed into individual wells of a 96-well plate. After 2 dpf, embryos are referred to as larvae. The larvae were monitored twice a day up to 93 hours post infection (hpi) and the number of dead larvae at each timepoint recorded. To confirm bacterial numbers in each injection, the same volume was ejected into 1 ml of phosphate buffered saline (PBS) and the viable counts determined on tryptic soy agar (TSA) plates. Survival curves were generated using GraphPad Prism.

Measurement of *S. aureus* growth in zebrafish. *S. aureus* cultures (3000 – 4000 CFU) were injected into the yolk sac circulation valley of zebrafish embryos at 30 hpf. At each timepoint until 5.2 dpf, five live larvae and any dead larvae, as well as 200 µl of E3 medium were transferred to 0.5 ml microcentrifuge tubes containing 1.4 mm ceramic beads. Each larva was homogenised using a FastPrep-24™ 5G Homogeniser and homogenates were serially diluted and plated to determine bacterial load.

Microinjection of morpholino-modified antisense oligonucleotides. One pmol of a morpholino-modified antisense oligonucleotide against the Pu.1 transcription factor [2] was injected into the yolk of one-cell stage zebrafish embryos, which were subsequently incubated at 28°C until injection with *S. aureus*. *S. aureus* cultures were injected into zebrafish embryos at 30 hpf. Larvae were maintained at 28°C, monitored twice a day up to 93 hpi (5.2 dpf) and the number of dead larvae at each timepoint recorded.

Quantification of (p)ppGpp. *S. aureus* strains were grown overnight in low-phosphate chemically defined media (CDM) [3] at 37°C. Cultures were diluted to an OD_{600} of 0.1 and grown for 2 hr prior to the addition of 3.7 MBq of $[^{32}P]H_3PO_4$ and incubation for a further 3 hr

at 37°C. Cultures were subsequently normalized for absorbance and suspended in 100 µl of 2 M formic acid. Cells were subjected to three freeze/thaw cycles and debris removed by centrifugation (17,000 × g for 5 min) before the lysate was filtered through a 3 kDa spin column. Ten µl were subsequently spotted on PEI-cellulose F thin-layer chromatography (TLC) plates (Merck Millipore), nucleotides separated, and TLC plates developed using a 1.5 M KH_2PO_4 pH 3.6 buffer. The radioactive spots were visualized using an FLA 7000 Typhoon PhosphorImager, and data were quantified using ImageQuantTL software.

Table S2. Bacterial strains used in this study

Strain	Relevant features	Reference
	***Escherichia coli* strains**	
XL1-Blue	Cloning strain: TetR	Stratagene
RMC0116	pCL55iTETr862 (iTET) in XL1-Blue: single-copy, integrative vector: CarbR	[4]
RMC0468	pCL55iTETr862-*rel* in XL1-Blue: CarbR	This study
RMC0469	pCL55iTETr862-*relP* in XL1-Blue: CarbR	This study
	***Staphylococcus aureus* strains**	
JE2 (WT)	CA-MRSA USA300 strain LAC derivative, lacking plasmids p01 and p03. Erm sensitive	[5]
RMC903	JE2 Δ*relQP* JE2 with in-frame deletions in *relQ*, and *relP*	[6]
RMC905	JE2 Δ*relQPA:* JE2 with in-frame deletions in *relQ*, *relP* and *rel*: ((p)ppGpp^0)	[6]
RMC1858	JE2 (p)ppGpp^0 iTET: CamR	This study
RMC1870	JE2 (p)ppGpp^0 iTET-*rel*: CamR	This study
RMC1871	JE2 (p)ppGpp^0 iTET-*relP*: CamR	This study
LAC*	CA-MRSA USA300 strain LAC derivative, lacking plasmid p03. Erm sensitive	[7]
LAC* Rel F128Y	LAC* with point mutation F128Y in the hydrolase domain of Rel	[8]

LAC* Rel_{syn}	LAC* with YQS deletion at amino acids 308-310 in the synthetase domain of Rel	[3]
NE1555	JE2 *codY*::Tn. Strain with transposon insertion in *codY*: ErmR	[5]
RMC1783	JE2 *codY*::Tn – transduced into fresh JE2: ErmR	This study
RMC2014	JE2 (p)ppGpp^0 *codY*::Tn: ErmR	This study

Antibiotics were used at the following concentrations - for *E. coli* cultures: carbenicillin (CarbR) 50-150 µg/ml. For *S. aureus* cultures: chloramphenicol (CamR) 7.5 µg/ml; erythromycin (ErmR) 10 µg/ml; Atet 50 ng/ml.

Table S3. Primers used in this study

Number	Name	Sequence
RMC165	F-AvrII-Rel	AAACCTAGGCCTAAATCATTGTTTAAGGCG
RMC166	R-SacII-Rel	AAACCGCGGCTAGTTCCAAACTCTTGTTACTG
RMC161	F-AvrII-RelP	AAACCTAGGTATCGGAGGTTAGTATAAAAATG
RMC162	R-SacII-RelP	AAACCGCGGCTACTCTGTTATTTCAGAATG

Restriction sites in primer sequences are underlined.

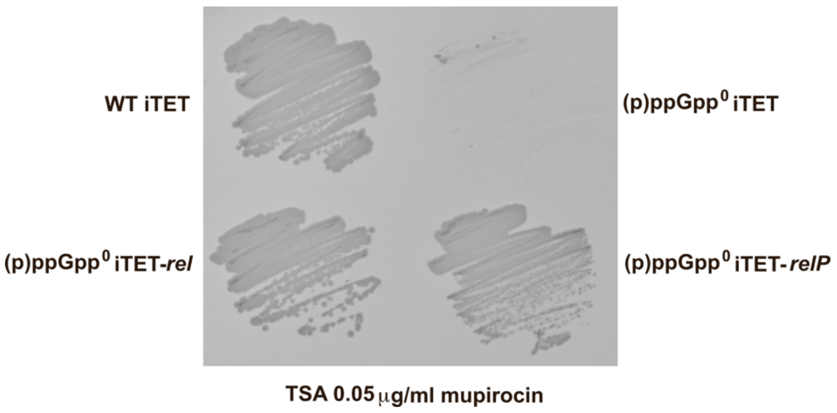

Fig S1. Characterisation of (p)ppGpp complementation strains. Strains were streaked on TSA plates containing 0.05 µg/ml mupirocin, which induces amino acid starvation. Leaky expression of either Rel or RelP from the pCL55iTET vector can complement the growth defect, indicative of (p)ppGpp production.

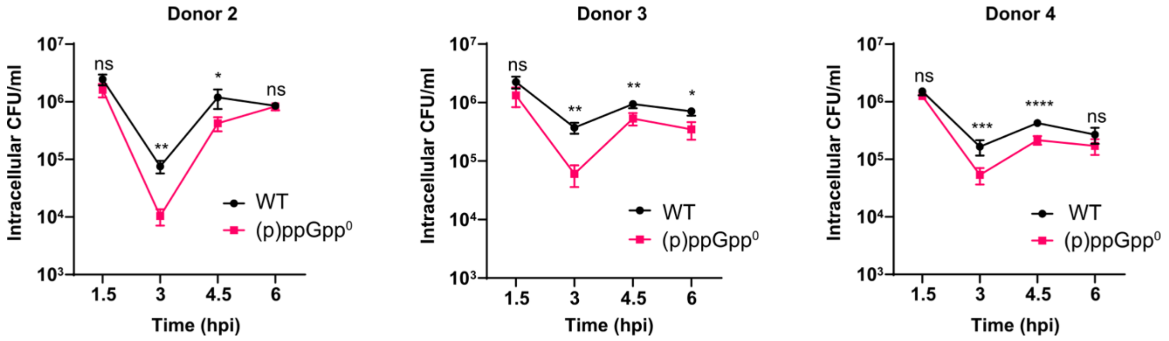

Fig S2. (p)ppGpp enhances *S. aureus* survival within primary human macrophages. Survival of WT and (p)ppGpp^0 mutant within human MDMs up to 6 hpi. MDMs were infected with bacteria at MOI 10 for 1 hr, before addition of 100 µg/ml gentamicin to kill extracellular bacteria. Infected MDMs were lysed at 1.5, 3, 4.5 or 6 hpi and plated to measure intracellular CFU/ml. Experiments were repeated four times (see also Fig 4B) using MDMs from four different donors. For each donor MDM population, two technical repeats were performed. Statistical significance was determined by unpaired *t*-test: ns, $P > 0.05$; * $P < 0.05$; ** $P < 0.01$; *** $P < 0.001$; **** $P < 0.0001$.

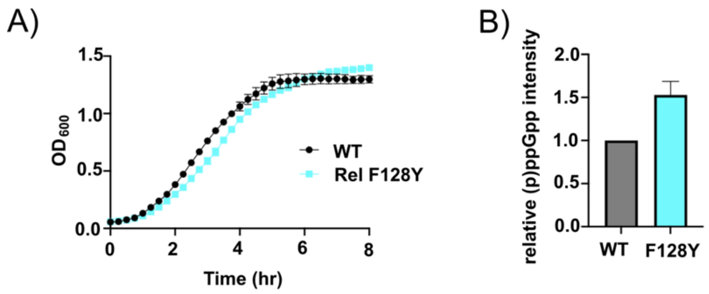

Fig S3. Characterisation of (p)ppGpp over-expression variant. A) Growth curve of WT and Rel F128Y mutant in nutrient replete media. Strains were diluted to an OD_{600} of 0.05 and growth monitored over 8 hrs. Average OD_{600} and standard deviation are plotted. **B)** Quantification of intracellular (p)ppGpp levels. Both strains were grown in the presence of ^{32}P-labeled H_3PO_4. The production of pppGpp and ppGpp was monitored by TLC and the radioactive spots quantified using ImageQuantTL. Average values and standard deviations from three independent experiments are shown.

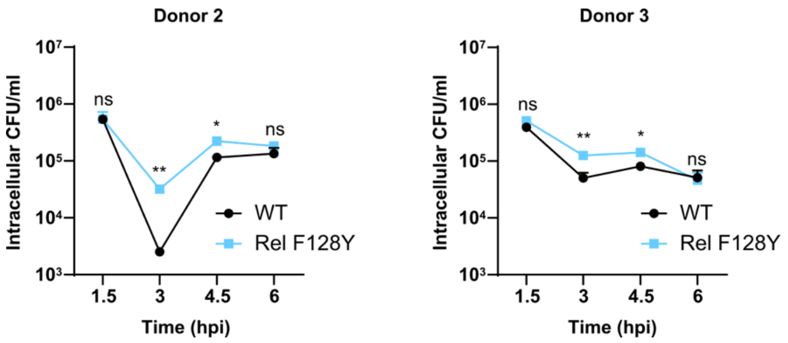

Fig S4. Overproduction of (p)ppGpp increases *S. aureus* survival in primary human macrophages. Survival of WT and Rel F128Y mutant within human MDMs up to 6 hpi. MDMs were infected with bacteria at MOI 10 for 1 hr, before addition of 100 µg/ml gentamicin to kill extracellular bacteria. Infected MDMs were lysed at 1.5, 3, 4.5 or 6 hpi and plated to measure intracellular CFU/ml. Experiments were repeated three times (see also Fig 4C) using MDMs from three different donors. For each donor MDM population, two technical repeats were performed. Statistical significance was determined by unpaired *t*-test: ns, $P > 0.05$; * $P < 0.05$; ** $P < 0.01$.

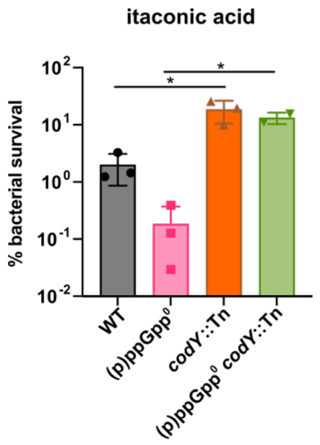

Fig S5. Deleting *codY* restores survival *in vitro*. Susceptibility of WT (black), (p)ppGpp^0 (pink), *codY*::Tn (orange) and (p)ppGpp^0 *codY*::Tn (light green) to 20 mM itaconic acid. Percentage bacterial survival with mean and standard deviation are plotted. Statistical analysis was performed using one-way ANOVA with Tukey's multiple comparisons test. * $P < 0.05$.

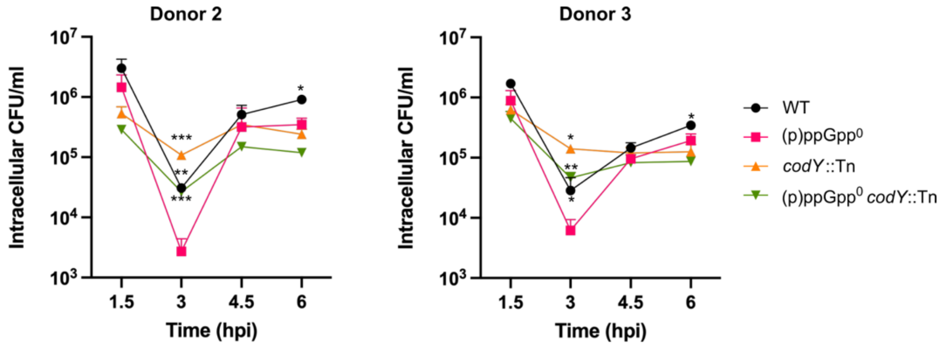

Fig S6. Deleting *codY* restores (p)ppGpp^0 survival within human macrophages. Intracellular survival of WT (black), (p)ppGpp^0 (pink), *codY*::Tn (orange) and (p)ppGpp^0 *codY*::Tn (light green) within primary human macrophages up to 6 hpi. MDMs were infected with bacteria at MOI 10 for 1 hr, before addition of 100 µg/ml gentamicin to kill extracellular bacteria. Infected MDMs were lysed at 1.5, 3, 4.5 or 6 hpi and plated to measure intracellular CFU/ml. Experiments were repeated three times (see also Fig 6C) using MDMs from three different donors. For each donor MDM population, two technical repeats were performed. Statistical significance was determined by unpaired t test: * $P < 0.05$; ** $P < 0.01$, *** $P < 0.001$.

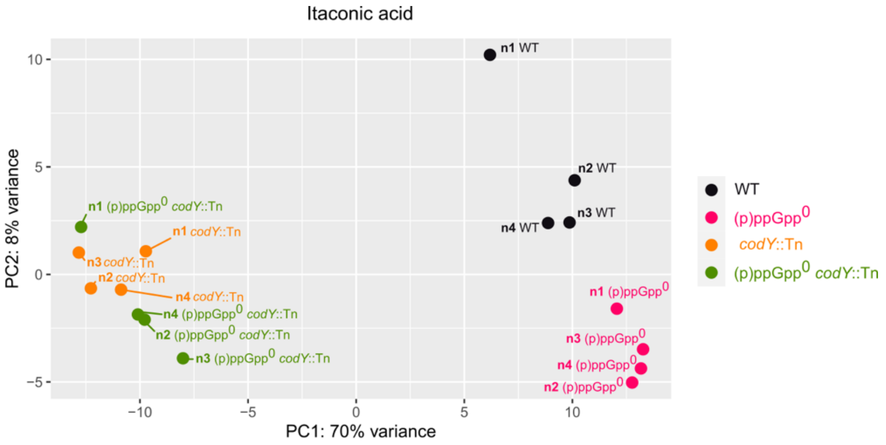

Fig S7. Presence or absence of the CodY transcriptional repressor is the largest contributor to variance within RNA-seq datasets for itaconic acid stress. Principal Component Analysis (PCA) of RNA-seq count data collected under itaconic acid-stress conditions for WT, (p)ppGpp^0, *codY*::Tn and (p)ppGpp^0 *codY*::Tn strains. PCA plot was generated by DESeq2 on the Galaxy web platform.

References

1. Nüsslein-Volhard C, Dahm R. Zebrafish: a practical approach. New York: Oxford University Press, **2002**.

2. Rhodes J, Hagen A, Hsu K, et al. Interplay of Pu.1 and gatal determines myelo-erythroid progenitor cell fate in zebrafish. Developmental Cell **2005**; 8:97-108.

3. Corrigan RM, Bowman L, Willis AR, Kaever V, Grundling A. Cross-talk between two nucleotide-signaling pathways in *Staphylococcus aureus*. J Biol Chem **2015**; 290:5826-39.

4. Corrigan RM, Campeotto I, Jeganathan T, Roelofs KG, Lee VT, Grundling A. Systematic identification of conserved bacterial c-di-AMP receptor proteins. Proc Natl Acad Sci U S A **2013**; 110:9084-9.

5. Fey PD, Endres JL, Yajjala VK, et al. A genetic resource for rapid and comprehensive phenotype screening of nonessential *Staphylococcus aureus* genes. mBio **2013**; 4:e00537-12.

6. Carrilero L, Urwin L, Ward E, et al. Stringent Response-Mediated Control of GTP Homeostasis Is Required for Long-Term Viability of *Staphylococcus aureus*. Microbiol Spectr **2023**:e0044723.

7. Boles BR, Thoendel M, Roth AJ, Horswill AR. Identification of genes involved in polysaccharide-independent *Staphylococcus aureus* biofilm formation. PLoS One **2010**; 5:e10146.

8. Bryson D, Hettle AG, Boraston AB, Hobbs JK. Clinical Mutations That Partially Activate the Stringent Response Confer Multidrug Tolerance in *Staphylococcus aureus*. Antimicrob Agents Chemother **2020**; 64.
